# Venom of the Annulated Sea Snake *Hydrophis cyanocinctus*: A Biochemically Simple but Genetically Complex Weapon

**DOI:** 10.3390/toxins13080548

**Published:** 2021-08-06

**Authors:** Hong-Yan Zhao, Yan Sun, Yu Du, Jia-Qi Li, Jin-Geng Lv, Yan-Fu Qu, Long-Hui Lin, Chi-Xian Lin, Xiang Ji, Jian-Fang Gao

**Affiliations:** 1 Hangzhou Key Laboratory for Animal Adaptation and Evolution, College of Life and Environmental Sciences, Hangzhou Normal University, Hangzhou 311121, China; zhaohy1000@hznu.edu.cn (H.-Y.Z.); sy@stu.hznu.edu.cn (Y.S.); linlh@hznu.edu.cn (L.-H.L.); 2 Hainan Key Laboratory of Herpetological Research, College of Fisheries and Life Science, Hainan Tropical Ocean University, Sanya 572022, China; yudu@hntou.edu.cn (Y.D.); lvjingeng@163.com (J.-G.L.); 3 MOE Key Laboratory of Utilization and Conservation for Tropical Marine Bioresources, Hainan Tropical Ocean University, Sanya 572022, China; 4 Jiangsu Key Laboratory for Biodiversity and Biotechnology, College of Life Sciences, Nanjing Normal University, Nanjing 210023, China; 181202118@njnu.edu.cn (J.-Q.L.); quyanfu@njnu.edu.cn (Y.-F.Q.); 5 College of Life and Environmental Sciences, Wenzhou University, Wenzhou 325035, China

**Keywords:** venom toxin, *Hydrophis cyanocinctus*, diversity, omics, positive selection

## Abstract

Given that the venom system in sea snakes has a role in enhancing their secondary adaption to the marine environment, it follows that elucidating the diversity and function of venom toxins will help to understand the adaptive radiation of sea snakes. We performed proteomic and de novo NGS analyses to explore the diversity of venom toxins in the annulated sea snake (*Hydrophis cyanocinctus*) and estimated the adaptive molecular evolution of the toxin-coding unigenes and the toxicity of the major components. We found three-finger toxins (3-FTxs), phospholipase A_2_ (PLA_2_) and cysteine-rich secretory protein (CRISP) in the venom proteome and 59 toxin-coding unigenes belonging to 24 protein families in the venom-gland transcriptome; 3-FTx and PLA_2_ were the most abundant families. Nearly half of the toxin-coding unigenes had undergone positive selection. The short- (i.p. 0.09 μg/g) and long-chain neurotoxin (i.p. 0.14 μg/g) presented fairly high toxicity, whereas both basic and acidic PLA_2_s expressed low toxicity. The toxicity of *H. cyanocinctus* venom was largely determined by the 3-FTxs. Our data show the venom is used by *H. cyanocinctus* as a biochemically simple but genetically complex weapon and venom evolution in *H. cyanocinctus* is presumably driven by natural selection to deal with fast-moving prey and enemies in the marine environment.

## 1. Introduction

Snakebite envenomation, as a neglected tropical disease, affects at least 1.8–2.7 million people, with upper estimates of 81,000–138,000 deaths annually in the world [1]. Many people are impressed with snakebite envenomation occurring on land, where most of the venomous snakes are, but they know little about the envenomation that occurs in the sea, largely due to low encounter rates with sea snakes and their less aggressive performance [2,3,4]. However, the snakebite envenomation caused by sea snakes should not be underestimated. As the largest group of marine reptiles, sea snakes are widely distributed in many tropical and subtropical waters across the Indo-Pacific Ocean [5,6], with relatively large population sizes in several regions [7,8,9,10,11]. There is no consensus on sea snake conservation around the world. Harvesting and trading of sea snakes, driven by the use of their skin, meat and blood for food, medicines and leather, are common in some Asian countries and regions [7,12,13] and over half of sea snakebites occur when fishermen handle sea snakes in fishing nets [14,15]. The neurotoxicity of sea-snake venoms is strong; thus, although the incidence of snakebites caused by sea snakes is relatively low, the death rate can be as high as 50% if the victims are not urgently treated with the correct steps [16,17].

Compared with their terrestrial relatives, sea snakes tend to simplify venom components to the extreme at the protein level [18]. Sea-snake venoms are mainly comprised of three-finger toxins (3-FTxs) and phospholipase A_2_ (PLA_2_) [19,20,21,22,23,24], with the short- and long-chain neurotoxins of 3-FTxs being the major and strong neurotoxic components [25,26]. Most sea snakes have to deal with fast-moving prey and, as such, the lethal neurotoxic venom can help them to quickly paralyze or kill prey almost instantaneously without letting them escape and avoid hurting themselves in the prey struggle [18]. Thus, from an evolutionary perspective, the venom system in sea snakes can enhance their secondary adaption to the marine environment. Recent studies on sea snakes of the genus *Hydrophis* show that venom toxins are far more diverse at the mRNA level than at the protein level [27,28,29]. Further elucidation of the diversity of venom toxins at both protein and mRNA levels would therefore facilitate our understanding of adaptive radiation of sea snakes, especially the taxonomic groups, such as the genus *Hydrophis*, with high speciation rates [30].

*Hydrophis cyanocinctus* used to be a dominant species in the southeast costal area of China and the South China Sea [11,31,32,33]. However, wide populations of *H. cyanocinctus* have declined rapidly due to overexploitation. Fortunately, all sea snakes that can be found in Chinese waters, including *H. cyanocinctus*, are in the newly published List of Key Protected Wild Animals in China as protected animals of national class II, meaning that China has banned the capture of sea snakes. In order to better understand and protect *H. cyanocinctus*, we need to know more about the diversity, evolution and function of its venom. The profiles of *H. cyanocinctus* venom at the omics level remain a sparsely studied area, with only two previous studies on the proteomic diversity of venom samples from Haikou, China [21], and Hara, Iran [19,21]. Here, we investigated the venom composition profile of *H. cyanocinctus* from the Xisha (Paracel) Islands in the South China Sea (Hainan, China) using a classical strategy for venomic analysis [34] and the toxin-coding genes profile in the venom-gland transcriptome with a de novo NGS technique. We also tested the positive selection based on the assembled toxin-coding genes with full CDS and determined the median lethal doses of both crude venom and some major venom components using ICR mice. Specifically, we aimed to clarify the diversity and correlation in abundance of venom toxins at both protein and mRNA levels, the strength of natural selection on venom toxins and the contribution of major venom components to the toxicity of the whole venom.

## 2. Results

### 2.1. Venomic Profile

**Figure 1:**
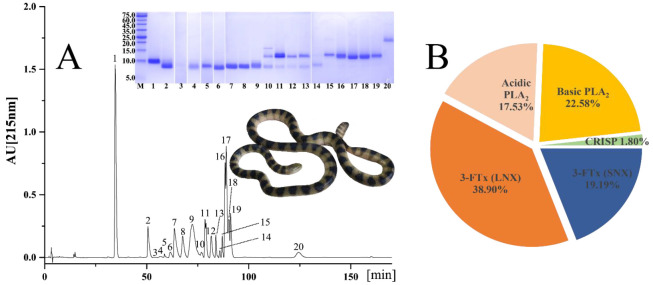
The venom proteomic profile of *H. cyanocinctus*. (**A**) Protein elution profile of venom. The proteins were separated by a C18 column as described in the Materials and Methods section. Fractions were collected and analysed by SDS-PAGE under reduced conditions. Protein bands were excised, tryptic-digested and analysed by nESI-MS/MS for their assignment to known protein families. (**B**) Relative abundance of venom toxin families. 3-FTx, three-finger toxin; LNX, long-chain α-neurotoxin; SNX, short-chain α-neurotoxin; PLA_2_, phospholipase A_2_; CRISP, cysteine-rich secretory protein. The details are listed in Table 1.

Twenty protein fractions (chromatographic peaks) were distinguished in venom by RP-HPLC and 26 protein bands from SDS-PAGE were identified by MS analysis (Figure 1A and Table 1). These protein bands could be classified into three toxin families: 3-FTx (58.09%), PLA_2_ (40.11%) and CRISP (1.80%) (Figure 1B and Table 1). Moreover, chromatographic peaks 1–9 were identified as 3-FTx and accounted for 54.94% of the total venom protein, peaks 10–19 were comprised of a high abundance of PLA_2_ (40.11%) and a relatively low abundance of 3-FTx (3.15%) and peak 20 contained only CRISP. Collectively, long-neurotoxin (LNX, 38.90%) was the predominant component in the 3-FTx family and short-neurotoxin (SNX, 19.19%) and acidic (17.53%) and basic (22.58%) PLA_2_s differed slightly from each other in expression abundance. Among four RP-HPLC factions of *H. cyanocinctus* venom, the SNX (i.p. 0.09 μg/g) and LNX (i.p. 0.14 μg/g) both presented fairly low LD_50_s for mice, whereas the basic and acidic PLA_2_s did not cause any death to the mice at the upper doses of 0.6 and 2.0 μg/g, respectively (Table 2). It is thus clear that the SNX is the most toxic component in *H. cyanocinctus* venom and that both SNX and LNX are far more toxic than PLA_2_s. Due to the high abundance of neurotoxins (SNX and LNX), *H. cyanocinctus* venom displayed relatively low LD_50_ for ICR mice (crude venom i.p. 0.26 μg/g and whole venom protein i.p. 0.16 μg/g). Another three major fractions (peaks 9, 16 and 17) were also determined to not cause death at a dose of 0.2 μg/g in mice; however, they were not further employed to determine the lethality with any other doses and thus are not displayed in the final results.

### 2.2. Transcriptome Assembly and Diversity of Toxin-Coding Unigenes

**Figure 2:**
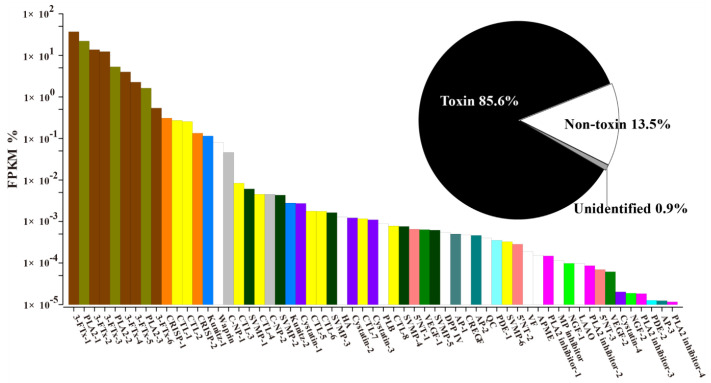
The venom-gland transcriptomic profile of *H. cyanocinctus*. The details are listed in the Supplementary Materials, Tables S1 and S2.

**Figure 3:**
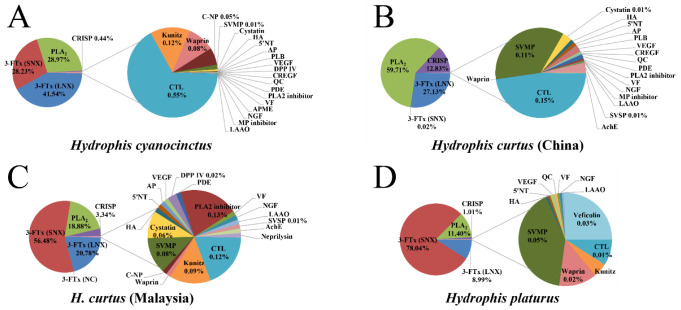
Comparison of the overall venom-gland transcriptomic profiles of three sea snakes: (**A**) current study; (**B**–**D**) cited and rearranged from Zhao et al. [24], Tan et al. [29] and Durban et al. [27], respectively. NC, non-conventional neurotoxin; SVSP, snake venom serine proteinase; AchE, acetylcholinesterase. Relative abundances less than 0.01% are not indicated in the toxin families.

In the present study, an Illumina Hiseq 2500 de novo NGS platform generated 109,660,888 pairs of raw reads from the venom-gland transcriptome, and 106,472,772 pairs of these data filtered as clean reads were assembled into 172,458 unigenes (N50/N90 = 1939/314) using Trinity. A total of 93,350 unigenes (N50/N90 = 2330/638) were clustered from these transcripts using Corset and 49,458 unigenes passing the quality filter (FPKM > 1) were used for the further analysis. These unigenes were finally categorized as toxins (59), non-toxins (37,405) and unidentified components (11,994), based on the similarity alignment against the NCBI NT/NR and Uniprot databases (strictly limited to “Serpentes”) using either BLAST or Diamond. Alternatively, the toxins, non-toxins and unidentified components accounted for 85.6%, 13.5% and 0.9% of the total abundance in FPKM of the *H. cyanocinctus* transcriptome (Figure 2 and Figure 3A). Compared with the non-toxins (8.42 FPKM/unigene), the toxins were expressed at an extremely high redundancy (159,248.25 FPKM/unigene). According to the annotated genes, 59 toxin-coding unigenes could be classified into 24 protein families (Figure 2 and Figure 3A and Supplementary Materials, Tables S1 and S2). The PLA_2_ (28.97%) and 3-FTx (69.76%) families constituted the most abundant components in the toxin-coding unigenes. The remaining 22 toxin families were expressed in low abundances with a total FPKM of 1.27%, including C-type lectin (CTL), CRISP, kunitz, waprin, C-type natriuretic peptide (C-NP), snake venom metalloproteinase (SVMP), cysteine-type inhibitor (cystatin), hyaluronidase (HA), 5′-nucleotidase (5′NT), aminopeptidase (AP), phospholipase B (PLB), vascular endothelial growth factor (VEGF), dipeptidyl peptidase 4 (DPP IV), cysteine-rich with EGF-like domain (CREGF), glutaminyl-peptide cyclotransferases (QC), phosphodiesterase (PDE), PLA_2_ inhibitor, venom factor (VF), acid phosphomonoesterase (APME), nerve growth factor (NGF), metalloproteinase inhibitor (MP inhibitor) and l-amino acid oxidase (LAAO). Tables S1 and S2 in the Supplementary Materials show detailed information on all the toxin-coding unigenes arranged according to protein family. Moreover, an overview of the venom-gland transcriptomic profiles revealed by de novo NGS in sea snakes in the current study and three recent studies is further presented in Figure 3.

### 2.3. Correlation between Translational and Transcriptional Abundances of Toxins

**Figure 4:**
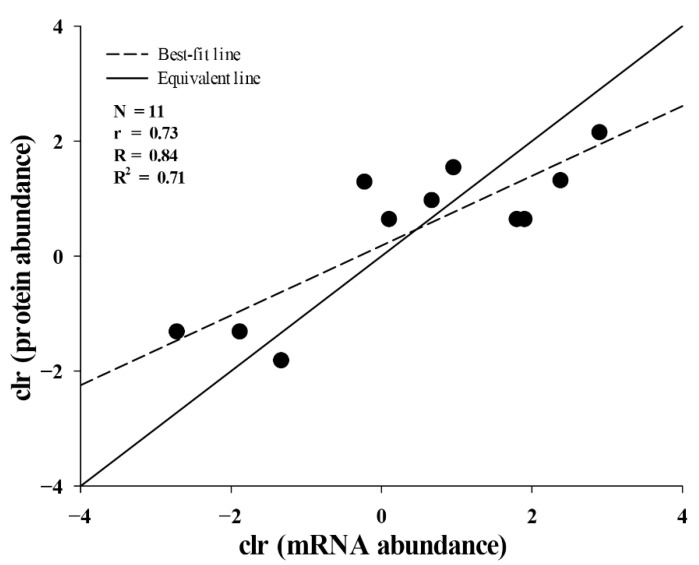
Correlation between mRNA and protein abundances of individual transcripts for each toxin family. *N*, number of toxin transcripts; *ρ*, Spearman’s rank correlation coefficient; *R*, Pearson’s correlation coefficient; *R^2^*, determination coefficient.

To evaluate the correlation between translational and transcriptional abundances of toxins, the MS data of protein bands were further assigned to 11 genes from an in-house database constructed from toxin-coding transcripts (Table 1). Considering that each of the five protein bands, including three 3-FTxs, one PLA_2_ and one CRISP, could be matched with 2–3 toxin-coding transcripts due to having the same scores, the abundance of each toxin at the protein level was divided equally among these transcripts, although this may have arbitrarily reflected the protein abundance translated by these transcripts. The results indicated that the abundance of these 11 toxins at the protein level was strongly correlated with that at the mRNA level (*ρ* = 0.73, *p* = 0.01) according to Spearman’s rank correlation analysis, and this was validated by the linear regression analysis with a relatively high Pearson’s correlation coefficient (*R* = 0.84, *p* = 0.001). Furthermore, the transcript abundances could explain the majority of variation in protein abundance (*R^2^* = 0.71, *p* = 0.001) (Figure 4).

### 2.4. Positive Selection in Evolutionary Adaption

Either the codeml or yn00 programs in PAML 4.8 were used to evaluate the positive selection in evolutionary adaption according to the full CDS homologs aligned to toxin-coding unigenes from *H. cyanocinctus*. Eighteen of the fifty-nine unigenes were excluded from the analysis due to a lack of full CDS homologs. Twenty-two codeml tests were performed on 27 full-length unigenes from 18 toxin families, with a significance level of 0.002 following Bonferroni correction (Table 3 and Table S3). Furthermore, the selection of 14 full-length unigenes from 10 toxin families together with their homologs was then directly analysed using yn00 (Table S4).

The 3-FTx (SNX), CRISP and NGF families were comprised of 2–3 unigenes and the sequence divergence in each family was lower than 10%. Thus, the unigenes from the same family were combined for codeml analysis with their homologs. The results rejected M1 (null hypothesis model) in favour of M2 (positive selection model), with all *p* < 0.05 after Bonferroni correction. For these tests, 28% (SNX), 22% (CRISP) and 15% (NGF) of codon sites exhibited positive selection of 4.74 ≤ *ω* ≤ 9.84. M0 showed that all sequence sites and branches in these unigenes exhibited an average strength of selection of 1.30 ≤ *ω* ≤ 2.79. The nucleotide sequence divergence between two PLA_2_ unigenes was >10%, and these unigenes were therefore analysed separately with their homologs. M1 could be easily rejected in favour of M2 with *p* < 0.001 following Bonferroni correction. Moreover, 32–38% of codon sites exhibited positive selection of 4.16 ≤ *ω* ≤ 8.00. M0 indicated that all the sequence sites and branches in these two PLA_2_ unigenes had an average strength of selection of 1.57 ≤ *ω* ≤ 1.95.

The nucleotide sequence divergence between two 5′NT unigenes was <10%, and these unigenes were therefore combined for positive selection analysis with their homologs. M1 could not be rejected with *p* = 0.12. M0 indicated that all the sequence sites and branches in these two unigenes had an average strength of selection of *ω* = 0.40. Three CTL unigenes presented relatively large divergences (>10%) in their nucleotide sequences; therefore, these unigenes were analysed separately with their homologs. However, M1 could not be rejected in these unigenes, with *p* > 0.05 in all cases. M0 indicated that all sequence sites and branches in three CTLs had an average strength of selection of 0.52 ≤ *ω* ≤ 0.62. Two kunitz unigenes were divided into two groups due to the large nucleotide sequence divergence. M1 could only be rejected in kunitz (2) in favour of M2, with *p* < 0.001 after Bonferroni correction. Thirty-eight percent of codon sites exhibited positive selection of *ω* = 7.95. M1 could not be rejected in kunitz (1), with *p* > 0.05 after Bonferroni correction. M0 showed that all the sequence sites and branches in these two unigenes had an average strength of selection of 0.33 ≤ *ω* ≤ 3.14.

For those unigenes subjected to condeml tests, six (cystatin, DPP IV, LAAO, PLB, SVMP and VF families) easily rejected M1, and they accepted M2 with *p* < 0.01 in all cases after Bonferroni correction. Additionally, 2–19% of codon sites were estimated to have positive selection of 3.82 ≤ *ω* ≤ 11.02. M0 indicated that all the sites and branches of the six unigenes had selection strengths of 0.43 ≤ *ω* ≤ 1.38. In contrast, tests for the AP, HA, PLA_2_ inhibitor, QC and VEGF unigenes could not reject M1, with all *p* > 0.05 after Bonferroni correction; M0 indicated a value for *ω* of 0.21–0.82.

Of the 14 remaining unigenes from 10 protein families, only one homolog with high similarity could be assigned to each sequence. In each pair of sequences, we found dN of 0–0.137 and dS of 0.009–0.347 for these unigenes from the venom-gland transcriptome. We calculated that dN/dS = 6.540, 3.673 and 4.251 for three LNX and 1.032 for one SVMP unigenes, from which we inferred that these sequences probably underwent positive selection. Moreover, 10 unigenes from eight protein families, including 5′NT, aminopeptidase, CREGF, cystatin, PDE, PLA_2_ inhibitor, MP inhibitor and waprin, were considered to exhibit purifying selection with dN/dS ranging from 0–0.529.

## 3. Discussion

As much attention has been paid to the composition of snake venom at the omics level, the strategy combining venomic analysis with de novo NGS analysis has been developed as an important means to unravel variation in snake venom and also provided an important reference for investigating the function of snake venom, the clinical symptoms of snakebites and the development of novel drugs. Here, we deployed a classic snake venomic workflow combined with RP-HPLC, SDA-PAGE and MS analyses [34] to decomplex the venomic profile of a pooled crude venom sample of *H. cyanocinctus*. With regard to the relative abundance of toxins at the protein level, 3-FTx and PLA_2_ could be defined as predominant toxin families, and this was similar to two previous venomic studies using samples from populations from Hara, Iran [19], and Haikou, Hainan, China [21]. However, the Xisha population exhibited the lowest abundance of 3-FTx but the highest abundance of PLA_2_ among the three populations and a comparatively low divergence between these two toxin families. Specifically, the expression of acidic and basic PLA_2_s was approximately 1.9- and 1.8-fold higher in the Xisha population than in the Haikou population, respectively. Furthermore, the ratio of SNX to LNX (1:2) in the Xisha population was opposite to that in the Hara (1.8:1) and Haikou (2.5:1) populations, and such an inconsistent divergence has also been found in *H. curtus* venoms [20,22,24]. This suggests that the potential symptoms of snakebites caused by *H. cyanocinctus* might vary among populations. Moreover, the lethality analysis indicated that the toxicity of *H. cyanocinctus* venom was largely determined by the 3-FTx family. The neurotoxins of *H. cyanocinctus* venom were less abundant in this study (58.09%) than in an earlier one using samples from the Hara population (81.1%; [19]).This finding explains why the venom from the Xisha population is less toxic than that from the Hara population (i.p. 0.172 μg/g). Importantly, the venom components with strong neurotoxicity in sea snakes have always been inferred to play an important role in paralyzing and killing fast-moving prey [18]; they may have evolved under the selective pressure imposed by such a diet in the marine environment. Thus, compared with the Hara population, the Xisha population might have experienced a regional diet with a relatively lower percentage of fast-moving prey and further evolved to express less abundant neurotoxins in its venom. However, this inference should be verified in further studies.

Of the studies carried out in the past decade to assess the diversity of toxin-coding genes at the venom-gland transcriptomic level in snakes, only four focused on sea snakes (*Acalyptophis peronii*, *H. curtus* and *Hydrophis platurus*) [27,28,29,35]. Compared with *H. curtus* (22 protein families, Figure 3B,C; [24,29]) and *H. platurus* (15 protein families, Figure 3D; [27]), *H. cyanocinctus* (24 protein families) displayed a relatively high diversity of toxin-coding unigenes from the venom-gland transcriptome. Similar to the venom-gland transcriptome reported previously for sea snakes [26,27,28,34], the PLA_2_ and 3-FTx families constituted the most abundant components in the toxin-coding unigenes of the *H. cyanocinctus* venom-gland transcriptome. However, the ratio of PLA_2_ to 3-FTx unigenes in abundance was 2.4:1 in *H. cyanocinctus*, which is significantly lower than that reported for *H. curtus* (4.1:1) [29] and *H. platurus* (7.6:1) [27]. Moreover, the ratio of SNX to LNX was 1:1.5 in *H. cyanocinctus*, which is extremely converse to the ratios reported for *H. curtus* (2.7:1) and *H. platurus* (8.7:1). However, whether this implies a lower neurotoxicity of venom in *H. cyanocinctus* (i.p. 0.26 μg/g crude venom in mice; current study) than in *H. curtus* (i.v. 0.20 μg/g; [20]) and *H. platurus* (i.p. 0.13 μg/g; [36]) needs to be further determined.

Concordance and discordance in venom compositions and abundance at the protein and mRNA levels always receive much scientific attention due to their role in uncovering the regulatory mechanisms at the protein/mRNA levels and the factors likely to affect the evolution and function of snake venom [37,38,39,40,41,42,43,44,45,46]. Our data show that *H. cyanocinctus* presents an apparent discordance in venom composition between protein (three major families) and mRNA (24 families). Due to the low abundance, some toxin families can easily be neglected at the protein level. Moreover, some toxin families with extremely low abundance might contribute little to the adaptive radiation of sea snakes and have even never been reported in closely related species. These toxin families can very likely be considered as pseudogenes that cannot be detected at the protein level. However, as our gene annotation was not based on a species-specific reference genome, whether the toxin-coding unigenes with low abundance and which are undetected at the protein level can be simply judged as pseudogenes or functional genes still needs to be verified. Concordance was verified in the abundance of 11 toxins at the protein and mRNA levels, suggesting that the post-transcriptional regulation might contribute little to the abundance of these toxins in *H. cyanocinctus*.

As a complicated biochemical arsenal, snake venom is believed to evolve to capture prey and attack potential enemies, thus comprising an ideal model to discuss the strength of natural selection exhibited by each toxin. Positive selection in the adaptive evolution of snake venom has been detected at both the individual toxin gene and venom-gland transcriptomic levels, with most studies conducted using site model analysis [28,47,48,49,50,51,52,53]. Here, considering that the power of the likelihood-ratio test might be reduced by the excessive sequence divergence [54], we divided the unigenes from the same toxin family into several groups at a threshold of 10% nucleotide sequence divergence and conducted codeml test separately with their homologs. Briefly, 17 of 27 toxin-coding unigenes from *H. cyanocinctus* were found to exhibit positive selection according to the likelihood ratio between M1 and M2, and this was verified by that between M7 and M8 (the results are not shown as they are nearly identical to those for M1 and M2). The percentage (63%, 17/27) of toxin-coding unigenes that underwent positive selection in *H. cyanocictus* was significantly lower than that for *Crotalus adamanteus* (89%, 24/27; [50]) and *H. curtus* (73%, 24/33; [24]), and it would even be much lower if the sequences analysed by yn00 were taken into account. Notably, all of the unigenes from the 3-FTx, PLA_2_ and CRISP families in *H. cyanocictus*, which could be detected in high abundance at the protein level and might have a practical function in predation and defence, were found to undergo positive selection. This finding suggests that the positive selection of toxin-coding unigenes in *H. cyanocictus* might be strongly driven by the fast-moving prey and enemies in the sea environment. Toxin-coding unigenes experiencing no positive selection might play no substantive role because *H. cyanocictus* has evolved to prefer a simplified diet consisting mainly of fish.

## 4. Conclusions

Here, we used an integrated omics strategy to investigate the diversity of venom toxins at the protein and mRNA levels in *H. cyanocinctus* from the South China Sea. We found an apparent discordance in venom composition between protein (three major families) and mRNA (24 families) and thus concluded that *H. cyanocinctus* uses venom as a biochemically simple but genetically complex weapon. The abundance of 11 toxins at the protein level was strongly correlated with that at the mRNA level, indicating that the post-transcriptional regulation contributes little to the abundance of these toxins. Nearly 51.2% (21 unigenes) of the toxin-coding unigenes with full lengths in *H. cyanocinctus* were found to have undergone positive selection, with the proportion being much lower than reported for other venomous snakes so far studied. Among four major venom components, the short-chain neurotoxin (SNX: i.p. 0.09 μg/g) and long-chain neurotoxin (LNX: i.p. 0.14 μg/g) of 3-FTx had fairly high toxicity (LD_50_) towards mice, whereas both basic and acidic PLA_2_s were not lethal to mice at the upper doses of 0.6 and 2.0 μg/g, respectively. Moreover, the crude venom and whole venom protein separately expressed toxicities of 0.26 and 0.16 μg/g toward mice. It is thus clear that the toxicity of *H. cyanocinctus* venom is largely determined by the 3-FTx family. For further recognition of the contribution of each venom component to the whole venom toxicity, the remaining RP-HPLC fractions should be employed to determine the lethality, especially for those with high abundance (e.g., peaks 9, 16 and 17).

## 5. Materials and Methods

### 5.1. Animals and Ethics

We collected six adults of the *H. cyanocinctus* species in the waters of the Xisha Islands in the South China Sea and transferred them to the Herpetological Research Center at Hainan Tropical Ocean University where they were maintained in a circulatory sea water system. The collection of snakes was approved by Hainan Tropical Ocean University (12 September 2017), and the experimental scheme was approved by the Animal Research Ethics Committee of Hangzhou Normal University (AREC2019109).

### 5.2. Isolation of Venom Proteins by RP-HPLC and SDS-PAGE

Fresh venom was extracted from each snake using a 100 μL plastic pipette, then centrifuged to remove impurities for 15 min at 10,000× *g* 4 °C, lyophilized, equally pooled and stored at −80 °C until use. Three milligrams of venom powder was re-dissolved in 0.1% TFA and centrifuged for 15 min at 10,000× *g*, 4 °C, and the supernatant was automatically loaded onto a Kromasil C18 column (250 × 4.6 mm, 5 μm particle size, 300 Å pore size; AkzoNobel, Bohus, Sweden) and separated at a flow rate of 1 mL/min using a Waters E600 HPLC system (Waters, Milford, MA, USA). The whole process was performed with a linear gradient of mobile phase A (0.1% TFA in water) and B (100% ACN): 0–15% B for 30 min, followed by 15–45% B for 120 min and 45–70% B for 20 min. Protein detection was monitored at 215 nm. The fractions were collected manually and concentrated in a Labconco CentriVap^®^ Centrifugal Concentrator (Labconco, Kansas, MO, USA). Protein concentration was determined according to Bradford [55]. The proteins of each fraction were separated by 18% SDS-PAGE under reduced conditions, and the gels were stained in 0.2% Coomassie Brilliant Blue R-250 and imaged using a Tanon Imaging system (Tanon Science & Technology, Shanghai, China).

### 5.3. Identification and Relative Abundance Estimation of Venom Proteins

Protein bands in the gels were excised and split into pieces with sizes of 1×1 mm, destained with 50 mM NH_4_HCO_3_ in 50% ACN and rinsed with 100% ACN in a ThermoMixer (ThermoFisher Scientific, Waltham, MA, USA). The proteins in gels were further treated with 50 mM DTT in 50 mM NH_4_HCO_3_ and alkylated with 0.14 M IAA in 50 mM NH_4_HCO_3_, rinsed again with 100% ACN in a ThermoMixer, and then were digested with trypsin (Promega, Madison, WI, USA) for 16 h at 37 °C. The digests (peptide mixture) were collected, lyophilized and re-dissolved in 0.1% TFA, desalted and enriched using an Acclaim™ PepMap™ 100 C18 column (Trap Cartridge; 5 × 0.3 mm, 5 μm; ThermoFisher Scientific), then separated by capillary RP-HPLC using an Acclaim™ PepMap™ RSLC 100 C18 column (NanoViper; 75 μm × 15 cm, 2 μm; ThermoFisher Scientific) with mobile phase A of 0.1% FA in water and B of 20% ACN in 0.1% FA as follows: 4% B for 3 min, 4–50% B for 47 min, 50–99% B for 4 min and 99% B for 6 min. Peptide eluents were subjected to a Q Exactive Orbitrap platform (ThermoFisher Scientific) according to the manufacturer’s instructions. The original MS/MS spectra were processed using Xcalibur software, and the sequence similarity was conducted using PEAKS X based on the UniProt database (strictly limited to “Serpentes”) or an in-house database (toxin transcripts from *H. cyanocinctus* venom-gland transcriptome). The mass tolerance was set at 0.1 Da; carbamidomethyl (C) and oxidation (M) were set as fixed and variable modifications, respectively.

The integration of the fractions in a chromatogram and the densitometry of the protein bands in an electropherogram were used to estimate the relative abundance of the venom composition [34,56]. Briefly, the relative abundance of each fraction was defined as the peak area measured by Empower software. When the fraction contained only one protein band, the relative abundance was directly defined from the peak area measurement, whereas for fractions containing more than one protein band, the relative abundance of each band was estimated by densitometry using Tanon-3500R software (Tanon Science & Technology, Shanghai, China).

### 5.4. Venom Gland cDNA Synthesis and Sequencing

After extraction of the venom, the snakes were kept in reptile pet terrariums and allowed to recover for four days to maximize the transcription in the venom glands, then sacrificed by injection of sodium pentobarbital (i.p. 100 mg/kg). Venom glands from both sides in each specimen were removed and dissected into pieces with sizes of < 2 mm × 2 mm, pooled and permeated with RNAlater (Qiagen, Hilden, Germany) at 4 °C overnight, and then stored at −80 °C until further use. Venom gland RNA of each specimen was extracted using TRIzol (Life Technologies, Carslbad, CA, USA), purified, concentrated and resuspended in 100 μL THE Ambion RNA storage solution (Life Technologies). The purity, integrity and concentration of RNA were evaluated using an Implen NanoPhotometer (Implen, München, Germany), Agilent 2100 Bioanalyzer (Agilent Technologies, Santa Clara, CA, USA) and Qubit 2.0 fluorometer (ThermoFisher Scientific), respectively. The mixture of total RNA for sequencing was prepared by equally mixing the RNA from the above six samples, in which the RNA integrity number (RIN) ranged from 7.4 to 8.0. Subsequently, mRNA was purified and enriched from the total RNA mixture using magnetic beads attached to oligo (dT), fragmented and then used as the template to synthesize a first-strand cDNA with random hexamer primers and reverse transcriptase (RNase H). After synthesis of the second-strand cDNA with dNTPs, RNase H and DNA polymerase I, the double-stranded cDNA was absorbed and purified using AMPure XP beads (Beckman Coulter), which was conducted with end repair and ligation of a poly (A) tail and adapters. The adapter-ligated fragments of 250–300 bp in length were preferentially screened, amplified by PCR and purified using AMPure XP beads to generate a final cDNA library. After assessment of the quality using an Agilent 2100 Bioanalyzer, the cDNA library was transferred to the Illumina HiSeq^TM^2500 platform (Illumina, San Diego, CA, USA) for high throughout sequencing.

### 5.5. Transcriptome Assembly, Annotation and Quantification

Prior to sequence assembly, the raw reads containing adapters and expressing low quality (Q ≤ 20) were eliminated. The clean reads were then assembled into contigs using Trinity [57] with the following steps: a k-mer dictionary (*k* = 25) was initially assembled from the different clean read sets and developed into a collection of linear contigs greedily searching using Inchworm; de Bruijn graphs were constructed using Chrysalis based on the contigs that shared at least one *k*–1-mer and the reads spanned the junction between contigs; the *de Bruijn* graphs with clean reads and paired-end reads were finally reconciled and the full-length transcripts for spliced isoforms and paralogs were reconstructed and arranged using Butterfly. The longest sequences with no redundancy derived from the transcripts in each gene locus by Corset [58] and CAP3 [59] were defined as unigenes and used as references for further analyses. Gene annotation was executed by searching against the NCBI NT/NR and UniProt protein databases (strictly limited to “Serpentes”). All clean reads were assigned to the reference unigenes using RSEM, and the number of reads matched to a given unigene was defined as the readcount and converted into FPKM for estimating the abundance of unigenes [60,61].

### 5.6. Detection of Positive Selection

Positive selection was detected on 42 transcripts with full-length CDS from 22 toxin families. Prior to analysis, the homologs of each transcript were gathered from the NCBI nucleotide database with a threshold of 10% divergence in sequences. The sequences were aligned by MAFFT 7.313 on the basis of the AA sequence. Signal peptides, gaps and stop codons were excluded from all analyses. Then, the best-fitting model for partitions was evaluated by PartitionFinder 2.1.1 [62] based on the Akaike information criterion (AIC) and a complete search. A maximum-likelihood phylogeny was constructed by IQ-TREE 1.6.8 in PhyloSuite 1.2.2 [63]; each operation was conducted by performing 5000 ultrafast bootstraps, and the SH-aLRT branch test was conducted by performing 1000 replicates. After confirmation that the chains were converged and mixed adequately, the maximum clade credibility tree was collected as the target tree.

Positive selection in nucleotide sites based on a likelihood-ratio test was evaluated using the codeml program in PAML 4.8 [64]. Generally, to evaluate whether the sites exhibited positive selection, the difference in log-likelihoods between models M1 (the null model refers to neutral selection with dN/dS = 1 and purifying selection with dN/dS < 1) and M2 (the positive model refers to positive selection with dN/dS > 1) was determined and compared to a χ^2^ distribution with two degrees of freedom. Then, the initial results were further verified by comparing models M7 and M8. A single ratio for all sites with average dN/dS was calculated by the M0 model. If only one homolog with complete CDS could be matched to our transcript, then dN and dS were directly calculated with the yn00 program in PAML 4.8, then the dN/dS value was calculated manually.

### 5.7. Lethality

Determination of the median lethal doses (LD_50_) was conducted by intraperitoneal injection of *H. cyanocinctus* venom and the major RP-HPLC fractions into ICR mice (22–26 g, *N* = 4; Laboratory Animal Center of Hangzhou Normal University) of either sex as previously described [65]. The mortality was recorded over 24 h, and LD_50_ was calculated using the Spearman–Karber method.

### 5.8. Statistical Analyses

The abundance of each toxin at the protein and mRNA levels was transformed by centred log-ratio (clr) as described by Rokyta et al. [66], and the correlation between the abundances of each toxin at both levels was assessed by three coefficients (Spearman’s rank correlation coefficient, Pearson’s correlation coefficient and determination coefficient), which were calculated through non-parametric correlation and linear regression analyses using Statistica 8.0 (StatSoft, Tulsa, OK, USA). The significance level was set at α = 0.05.

## Figures and Tables

**Table 1:** Assignment of the chromatographic fractions and electrophoretic bands from *H. cyanocinctus* venom to protein families.

Peak	%	MW (kDa)	Peptide Ion		MS/MS-Derived Sequence	Protein Family/Species/Accession/Transcript ID
			*m*/*z*	*z*		
1	19.19	9.3	775.3	2	TTTNCAESSCYKK	3-FTx (SNX); *Hydrophis cyanocinctus*; P25494; Hcy|29338, Hcy|29549, Hcy|28274
2	3.74	7.3	537.3 727.3	3 2	XEFGCAATCPTVBR XEFGCAATCPTVB	3-FTx (LNX); *Laticauda colubrina*; P0C8R8; Hcy|29273
3	0.27	7.3	537.3 727.3	3 2	XEFGCAATCPTVBR XEFGCAATCPTVB	3-FTx (LNX); *L. colubrina*; P0C8R8; Hcy|29273
4	0.22	7.3	805.4 727.3	2 2	XEFGCAATCPTVBR XEFGCAATCPTVB	3-FTx (LNX); *L. colubrina*; P0C8R8; Hcy|29273
5	0.23	7.4	805.4 727.3	2 2	XEFGCAATCPTVBR XEFGCAATCPTVB	3-FTx (LNX); *L. colubrina*; P0C8R8; Hcy|29273
6	1.48	7.0	537.3 727.3	3 2	XEFGCAATCPTVBR XEFGCAATCPTVB	3-FTx (LNX); *L. colubrina*; P0C8R8; Hcy|29273
7	6.17	7.3	531.9 479.9 623.2	3 3 2	RXEMGCAATCPTVB XEMGCAATCPTVB SWCDAFCGSR	3-FTx (LNX); *Hydrophis hardwickii*; Q8UW28; Hcy|29273
8	5.85	7.3	588.3 580.3 495.8	2 4 2	GBVXEXGCTAB THPYBPETCPPGBNXCYBB VXEXGCTAB	3-FTx (LNX); *H. hardwickii*; A3FM53; Hcy|29273
9	17.79	7.6	588.3 638.2 788.4 580.2	2 2 2 4	GBVXEXGCTAB SWCDAFCSSR VXEXGCTABCPTVB THPYBPETCPPGBNXCYBB	3-FTx (LNX); *H. hardwickii*; A3FM53; Hcy|29273, Hcy|29140
10	1.22	17.2	591.3 646.0 822.3	3 3 2	BVCDCDVAAAECFAR NXVBFSYVXTCANHNR VCDCDVAAAECFAR	Basic PLA_2_; *Hydrophis schistosus*; P00610; Hcy|29208
		11.8	591.3 646.0 548.6	3 3 3	BVCDCDVAAAECFAR NXVBFSYVXTCANHNR VCDCDVAAAECFAR	Basic PLA_2_; *H. schistosus*; P00610; Hcy|29208
	0.77	7.3	588.3 587.6 495.8 638.2	2 3 2 2	GBVXEXGCTAB GBVXEXGCTABCPTVB VXEXGCTAB SWCDAFCSSR	3-FTx (LNX); *H. hardwickii*; Q8UW29; Hcy|29273
11	6.19	11.7	618.8 821.9	2 2	BVCDCDVAAAB VCDCDVAAABCFAR	Basic PLA_2_; *H. hardwickii*; Q8UW30; Hcy|29208
		7.3	591.3 548.6 646.0	3 3 3	BVCDCDVAAAECFAR VCDCDVAAAECFAR NXVBFSYVXTCANHNR	Basic PLA_2_; *H. schistosus*; P00610; Hcy|29208
12	3.35	11.7	591.3 548.6 757.8 646.0 446.2	3 3 2 3 3	BVCDCDVAAAECFAR VCDCDVAAAECFAR NAYNNANYNXDTB NXVBFSYVXTCANHNR XHDDCYGEAEB	Basic PLA_2_; *H. schistosus*; P00610; Hcy|29208
	0.49	7.4	588.3 495.8	2 2	GBVXEXGCTAB VXEXGCTAB	3-FTx (LNX); *H. hardwickii*; Q8UW29; Hcy|29273
13	1.51	12.0	822.3 591.3	2 3	VCDCDVAAAECFAR BVCDCDVAAAECFAR	Basic PLA_2_; *H. schistosus*; P00610; Hcy|29208
	0.80	7.3	588.3 638.2 587.6	2 2 3	GBVXEXGCTAB SWCDAFCSSR GBVXEXGCTABCPTVB	3-FTx (LNX); *H. hardwickii*; A3FM53; Hcy|29273
14	0.29	12.0	591.3 548.6	3 3	BVCDCDVAAAECFAR VCDCDVAAAECFAR	Basic PLA_2_; *H. schistosus*; P00610; Hcy|29123
	1.10	7.7	773.4 588.3 587.6 495.8 638.2	3 2 3 2 2	THPYBPETCPPGBNXCYBB GBVXEXGCTAB GBVXEXGCTABCPTVB VXEXGCTAB SWCDAFCSSR	3-FTx (LNX); *H. hardwickii*; A3FM53; Hcy|29273, Hcy|29296
15	2.05	12.3	455.2	2	TAAXCFAR	Acidic PLA_2_; *Tropidechis carinatus*; Q45Z26; Hcy|29123
16	7.15	11.4	455.2 644.3	2 3	TAAXCFAR DNNDECBAFXCNCDR	Acidic PLA_2_; *T. carinatus*; Q45Z28; Hcy|29123
17	10.02	10.4	455.8 687.7	2 3	XTXYSWB CFABAPYNNBNYNXDTB	Basic PLA_2_; *Austrelaps superbus*; Q9PUH5; Hcy|29057
18	3.21	10.7	644.3 455.2	3 2	DNNDECBAFXCNCDR AFXCNCDR	Acidic PLA_2_; *Notechis scutatus scutatus*; Q9PSN5; Hcy|29123
19	5.11	11.4	455.3 601.8 528.2	2 2 2	TAAXCFAR GGSGTPVDEXDR AFXCNCDR	Acidic PLA_2_; *N. s. scutatus*; Q9PSN5; Hcy|29057, Hcy|29123
20	1.80	23.6	448.2	3	CTFAHSPEHTR	CRISP; *H. hardwickii*; Q8UW11; Hcy|29512, Hcy|29717

X: Leu/Ile; B: Lys/Gln. 3-FTx, three-finger toxin; PLA_2_, phospholipase A_2_; CRISP, cysteine-rich secretory protein; LNX, long-chain α-neurotoxin; SNX, short-chain α-neurotoxin. Transcripts are listed in the Supplementary Materials, Table S1.

**Table 2:** The median lethal doses (LD_50_) of *H. cyanocinctus* venom and the major fractions separated by RP-HPLC.

Fraction	Toxin	Intraperitoneal LD_50_ (μg/g) *
-	Crude venom	0.26 (0.23–0.30)
-	Whole venom protein **	0.16 (0.12–0.20)
1	Short neurotoxin	0.09 (0.07–0.12)
7	Long neurotoxin	0.14 (0.09–0.21)
11	Basic PLA_2_	>0.6
18	Acidic PLA_2_	>2.0

*: values in parentheses are 95% confidence limits. **: Whole venom protein was defined as the protein in crude venom and quantified by the crude venom after determination of the protein concentration.

**Table 3:** Summary of codeml tests for positive selection of toxins from venom-gland transcriptome in *H. cyanocinctus*.

Toxins (No.)	M1: Nearly Neutral			−ln*L*	M2: Positive Selection				−ln*L*	M0: *ω*	Δ ^a^	*p*-Value ^b^
3-FTx (1, 2, 3)	*p*:	0.56	0.44	608.01	*p*:	0.45	0.27	0.28	588.51	2.79	39.00	3.40 × 10^−9^ *
	*ω*:	0.00	1.00		*ω*:	0.00	1.00	9.84				
5NT (1, 2)	*p*:	0.60	0.40	3831.74	*p*:	0.90	0.00	0.10	3829.66	0.40	4.16	0.12
	*ω*:	0.00	1.00		*ω*:	0.21	1.00	2.57				
AP (1)	*p*:	0.63	0.37	5224.95	*p*:	0.93	0.00	0.07	5221.58	0.40	6.74	0.03
	*ω*:	0.00	1.00		*ω*:	0.22	1.00	3.95				
CRISP (1, 2)	*p*:	0.47	0.53	2499.04	*p*:	0.40	0.39	0.22	2458.11	1.38	81.87	0.00*
	*ω*:	0.00	1.00		*ω*:	0.00	1.00	4.74				
CTL (1)	*p*:	0.56	0.44	1046.59	*p*:	0.82	0.00	0.18	1045.29	0.52	2.60	0.27
	*ω*:	0.00	1.00		*ω*:	0.19	1.00	2.49				
CTL (2)	*p*:	0.60	0.40	1177.31	*p*:	0.80	0.07	0.13	1172.58	0.56	9.46	8.83 × 10^−3^
	*ω*:	0.06	1.00		*ω*:	0.22	1.00	3.07				
CTL (3)	*p*:	0.55	0.45	1186.50	*p*:	0.60	0.00	0.40	1184.23	0.62	4.55	0.10
	*ω*:	0.00	1.00		*ω*:	0.00	1.00	1.65				
Cystatin (1)	*p*:	0.29	0.71	979.80	*p*:	0.96	0.00	0.04	969.73	1.15	20.13	4.25 × 10^−5^ *
	*ω*:	0.04	1.00		*ω*:	0.84	1.00	11.02				
DPP IV	*p*:	0.64	0.36	4740.05	*p*:	0.83	0.11	0.06	4730.48	0.43	19.12	7.05 × 10^−5^ *
	*ω*:	0.00	1.00		*ω*:	0.15	1.00	3.82				
HA	*p*:	0.45	0.55	2976.76	*p*:	0.52	0.00	0.48	2975.36	0.62	2.81	0.25
	*ω*:	0.00	1.00		*ω*:	0.00	1.00	1.38				
Kunitz (1)	*p*:	0.82	0.18	1502.79	*p*:	0.82	0.11	0.08	1502.79	0.33	0.00	1.00
	*ω*:	0.21	1.00		*ω*:	0.21	1.00	1.00				
Kunitz (2)	*p*:	0.44	0.56	944.01	*p*:	0.18	0.44	0.38	916.66	3.14	54.70	1.32 × 10^−12^ *
	*ω*:	0.05	1.00		*ω*:	0.00	1.00	7.95				
LAAO	*p*:	0.44	0.56	6029.83	*p*:	0.39	0.43	0.17	5970.56	1.03	118.53	0.00*
	*ω*:	0.00	1.00		*ω*:	0.00	1.00	4.00				
NGF (1, 2)	*p*:	0.51	0.49	1366.53	*p*:	0.39	0.46	0.15	1342.54	1.30	47.98	3.81 × 10^−11^ *
	*ω*:	0.07	1.00		*ω*:	0.13	1.00	5.87				
PLA_2_ (1)	*p*:	0.47	0.53	1913.94	*p*:	0.41	0.21	0.38	1877.93	1.57	72.01	2.22 × 10^−16^ *
	*ω*:	0.01	1.00		*ω*:	0.00	1.00	4.16				
PLA_2_ (2)	*p*:	0.45	0.55	808.89	*p*:	0.68	0.00	0.32	796.33	1.95	25.12	3.51 × 10^−6^ *
	*ω*:	0.00	1.00		*ω*:	0.15	1.00	8.00				
PLA_2_ inhibitor (1)	*p*:	0.48	0.52	1900.98	*p*:	0.50	0.00	0.50	1900.96	0.50	0.04	0.98
	*ω*:	0.00	1.00		*ω*:	0.00	1.00	1.07				
PLB	*p*:	0.56	0.44	3750.75	*p*:	0.53	0.45	0.02	3742.03	0.54	17.44	1.63 × 10^−4^ *
	*ω*:	0.00	1.00		*ω*:	0.00	1.00	6.82				
QC	*p*:	0.78	0.22	2194.10	*p*:	0.79	0.00	0.21	2194.09	0.21	0.01	1.00
	*ω*:	0.00	1.00		*ω*:	0.00	1.00	1.05				
SVMP (1)	*p*:	0.48	0.52	8066.12	*p*:	0.35	0.46	0.19	7910.53	1.38	311.17	0.00 *
	*ω*:	0.04	1.00		*ω*:	0.00	1.00	5.06				
VEGF	*p*:	0.31	0.69	1101.80	*p*:	0.87	0.00	0.13	1101.01	0.82	1.58	0.45
	*ω*:	0.00	1.00		*ω*:	0.49	1.00	3.83				
VF	*p*:	0.53	0.47	13764.79	*p*:	0.51	0.46	0.04	13,726.91	0.61	75.76	0.00 *
	*ω*:	0.00	1.00		*ω*:	0.00	1.00	4.96				

*, significance at the 5% level after Bonferroni correction; ^a^, negative twice the difference in ln*L* between M1 and M2; ^b^, *p*-value before correction.

## Data Availability

The venom-gland transcriptome raw data can be found in the NCBI Sequence Read Archive (SRA) under the accession number PRJNA736827.

## Supplementary Materials

The following are available online at https://www.mdpi.com/article/10.3390/toxins13080548/s1, Table S1. Amino acid sequences translated from toxin unigenes in the venom-gland transcriptome of the annulated sea snake (*Hydrophis cyanocinctus*) from the South China Sea. Sequences with partial CDS are indicated in italics; Table S2. FPKM% of protein family in the venom-gland transcriptome of the annulated sea snake (*Hydrophis cyanocinctus*) from the South China Sea; Table S3. Sequences used for positive selection detection in the codeml test; Table S4. Sequences used for positive selection detection in the yn00 test.
